# Supplementary material for: Peptidoglycan recruitment by a penicillin binding protein
Source: Nat Commun. 2025 Dec 17;16:11244. doi: 10.1038/s41467-025-66095-y (PMC12717099; doi:10.1038/s41467-025-66095-y)

Supplementary Information for

Peptidoglycan recruitment by a Penicillin Binding Protein

Yamanappa Hunashal[1,^,$], Matthieu Fonvielle[2,^,#], Masumi Takayama Kobayashi[1], Meng S. Choy[1], Ganesan Senthil Kumar[1,3], Paul Ugalde Silva[4,~], Yucheng Liang[2], Charlene Desbonnet[4], Louis B. Rice[4], Michel Arthur[2], Rebecca Page[5] & Wolfgang Peti[1,*]

[1]Department of Molecular Biology and Biophysics, University of Connecticut Health, Farmington, USA; [2]INSERM ERL 1336, UMRS 8228, Sorbonne Université-ENS-PSL-CNRS, Paris, F-75006, France; [3]National Institute of Immunology, New Delhi, India; [4]Department of Medicine, Rhode Island Hospital, Warren Alpert Medical School of Brown University, Providence, USA; [5]Department of Cell Biology, University of Connecticut Health, Farmington, USA.

[^]contributed equally

[$]Current address: Division of Science, New York University Abu Dhabi, Abu Dhabi, PO Box 129188, United Arab Emirates

[#]Current address: Institute for Integrative Biology of the Cell (I2BC), CEA, CNRS, Université Paris-Saclay, 91198 Gif-sur-Yvette, France

~Current address: Department of Microbiology and Infectious Diseases, Faculty of Medicine and Health Sciences, Université de Sherbrooke, Sherbrooke, Québec, Canada

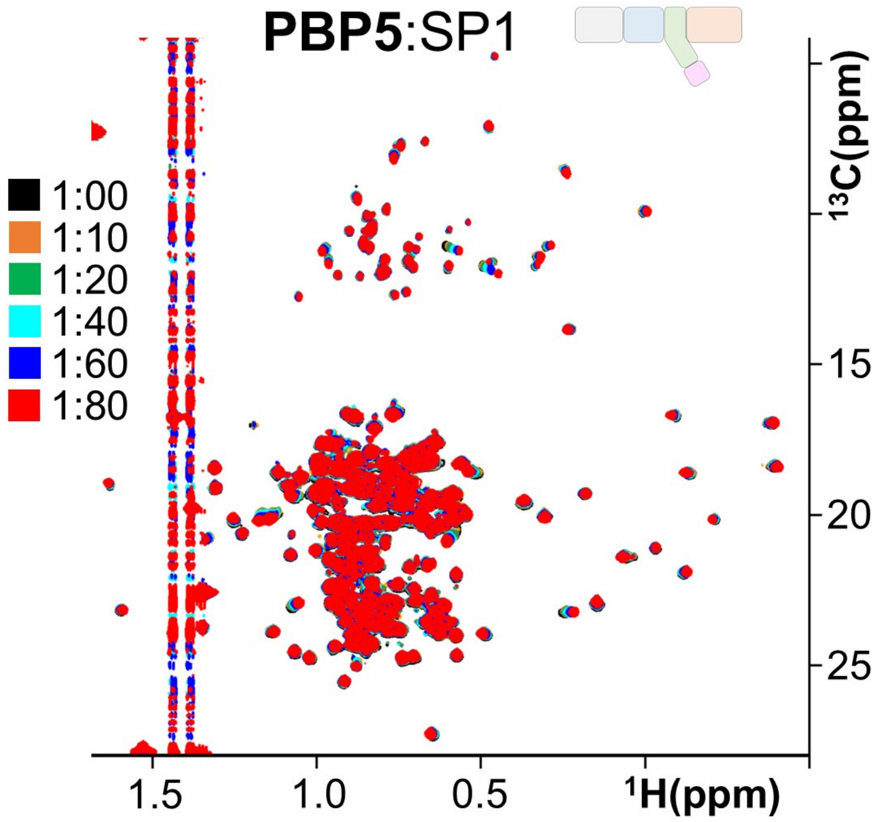

Supplementary Figure 1: Interaction of the *E. faecium* STEM peptide (SP1) with PBP5. Overlay of 2D [^{1}H,^{13}C] HMQC spectrum of PBP5 (black) with increasing concentrations of SP1 (PBP5:SP1, up to 1:80 ratio; orange to red).

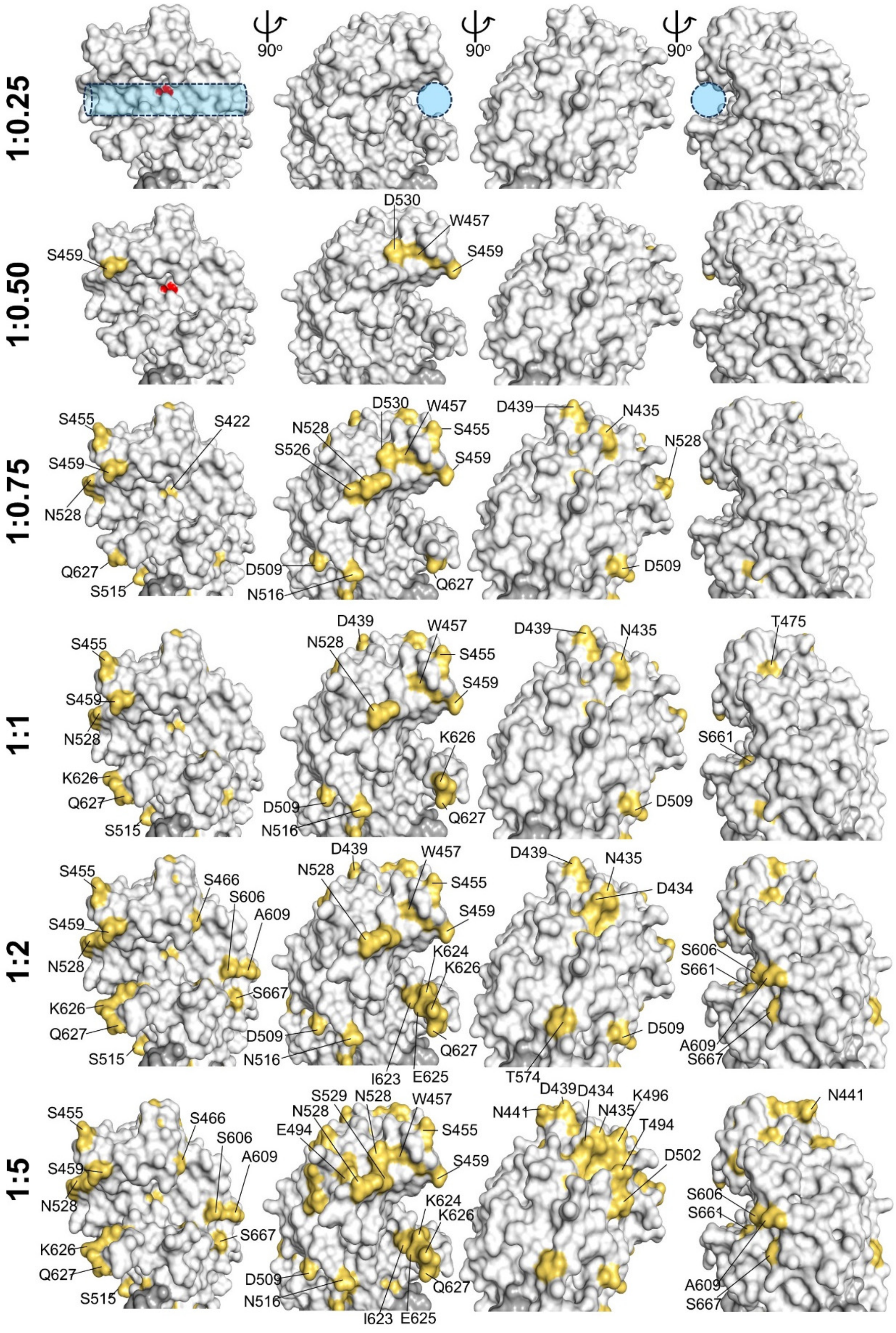

Supplementary Figure 2: PG NMR interaction study. Mapping of disappearing N/H^N cross peaks/PBP5 residues (yellow) with increasing amount of reduced PG. Ratio (PBP5:reduced PG) indicated on the right.

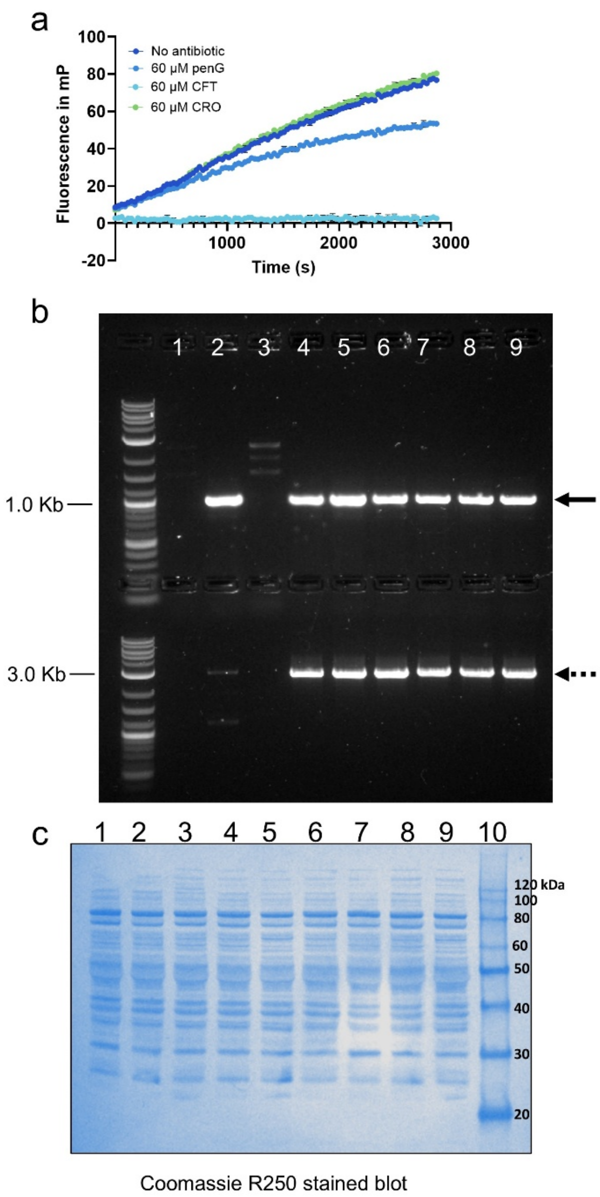

Supplementary Figure 3: PG formation. (**a**) Ceftriaxone does not bind/inhibit PBP5. BOCILLIN FL fluorescence polarization assay with PBP5 (blue) and penicillin (PenG; light blue), ceftaroline (CFT, teal) and ceftriaxone (CRO, green). All three antibiotics were used at a fixed 60 μM concentration. n = 3; average ± std deviation. (**b**) Colony PCR of *E. faecium* D344SRF. *Top*: Amplification from *ftsW* downstream into the beginning of *pbp5*; Lane 1, *E. faecium* D344SRF; Lane 2, *E. faecium* D344SRF + PBP5 plasmid; Lane 3, *E. faecium* D344SRF + empty plasmid; Lane 4, *E. faecium* D344SRF + PBP5_V1 plasmid; Lane 5, *E. faecium* D344SRF + PBP5_V2 plasmid; Lane 6, *E. faecium* D344SRF + PBP5_V3 plasmid; Lane 7, *E. faecium* D344SRF + PBP5_V4 plasmid; Lane 8, *E. faecium* D344SRF + PBP5_V5 plasmid; Lane 9, *E. faecium* D344SRF + PBP5_V6 plasmid. Solid arrow indicates expected size of 1009 bp. *Bottom*: Amplification from *ftsW* (reverse read). Dashed arrow indicates expected size of 2939 bp. (**c**) PBP5 protein expression; Commassie R250 stain. Lane numbers correspond to experiments in **b**; Lane 10: Molecular weight marker.

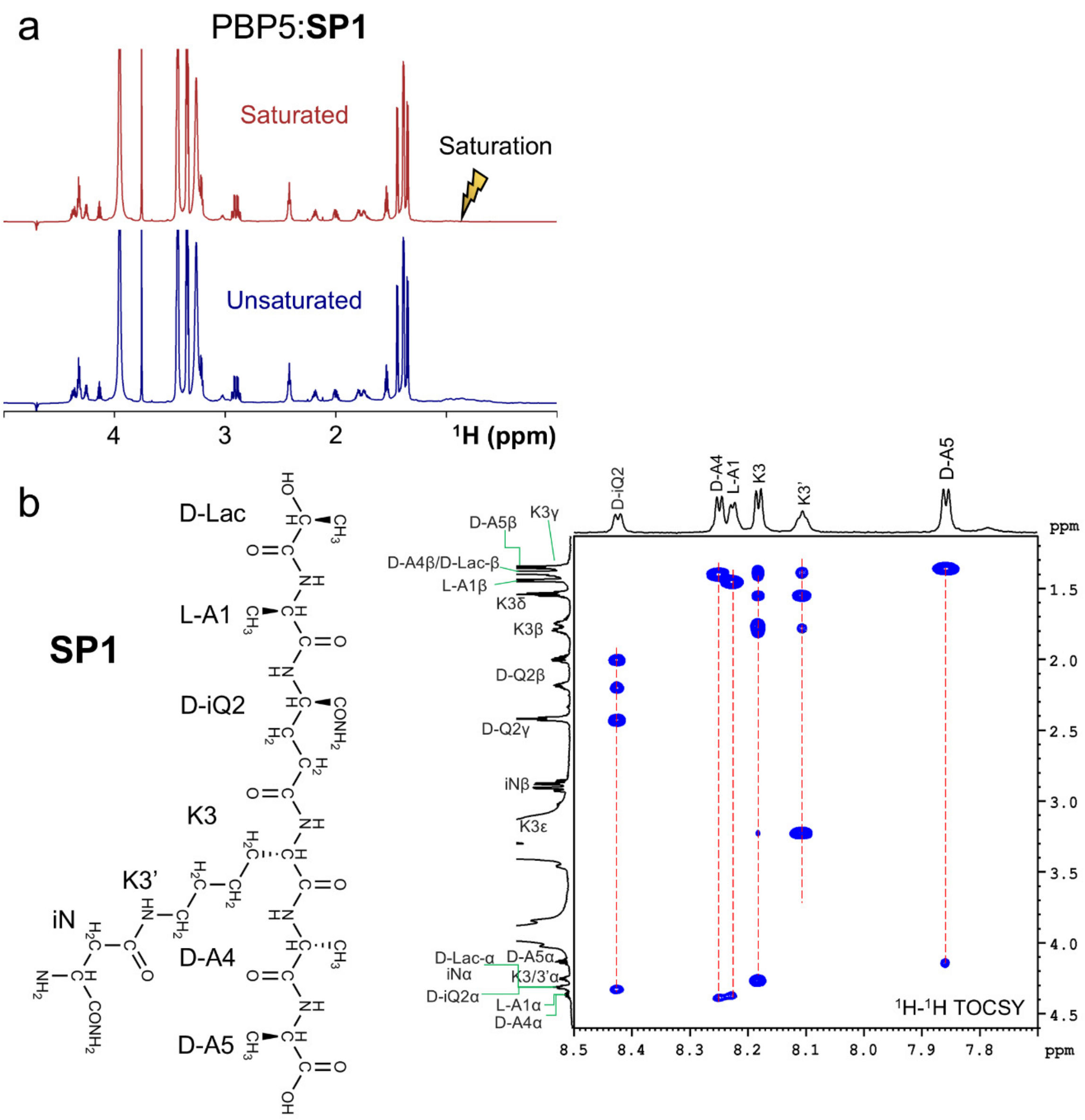

Supplementary Figure 4: Interaction of the *E. faecium* STEM peptide (SP1) with PBP5. (**a**) Overlay of 1D ^{1}H saturation transfer difference (STD) NMR spectrum of PBP5 and SP1 at 1:100 ratio; red, saturated (0.73 ppm); blue, reference (unsaturated, -40 ppm). (**b**) 2D [^{1}H,^{1}H] TOCSY spectrum of SP1 with the resonance assignments, chemical structure shown left.

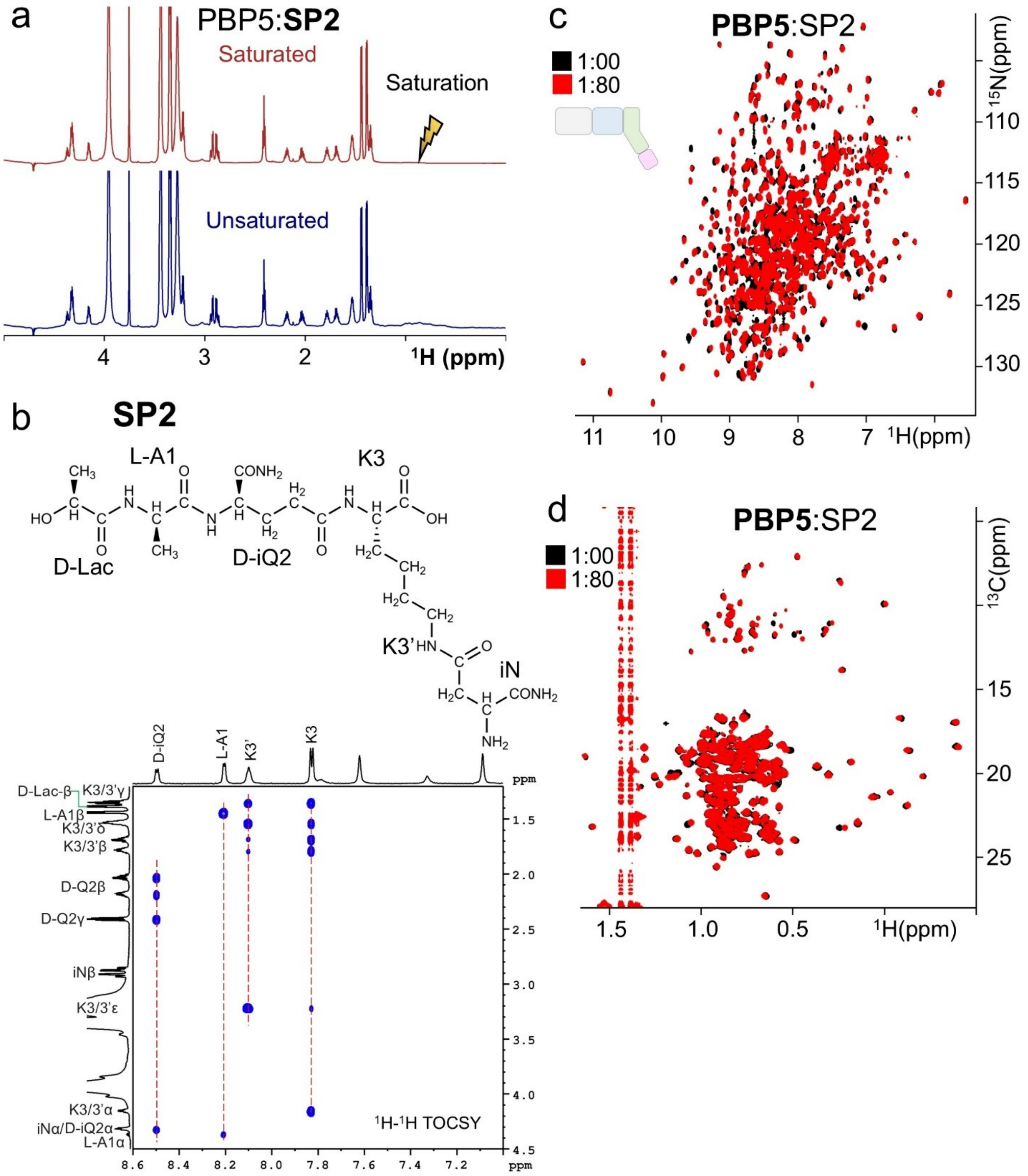

Supplementary Figure 5: D-Ala-D-Ala does not contribute to the PBP5 interaction. (**a**) Overlay of saturation transfer difference (STD) NMR spectrum of PBP5 and SP2 at 1:100 ratio; red is saturated (0.73 ppm), and blue is reference (unsaturated, -40 ppm) spectrums. (**b**) 2D [^{1}H,^{1}H] TOCSY spectrum of SP2 with the resonance assignments, chemical structure shown above. Overlay of 2D (**c**) [^{1}H,^{15}N] TROSY and (**d**) [^{1}H,^{13}C] HMQC spectrum of PBP5 (black) with SP2 (red) at 1:80 ratio.

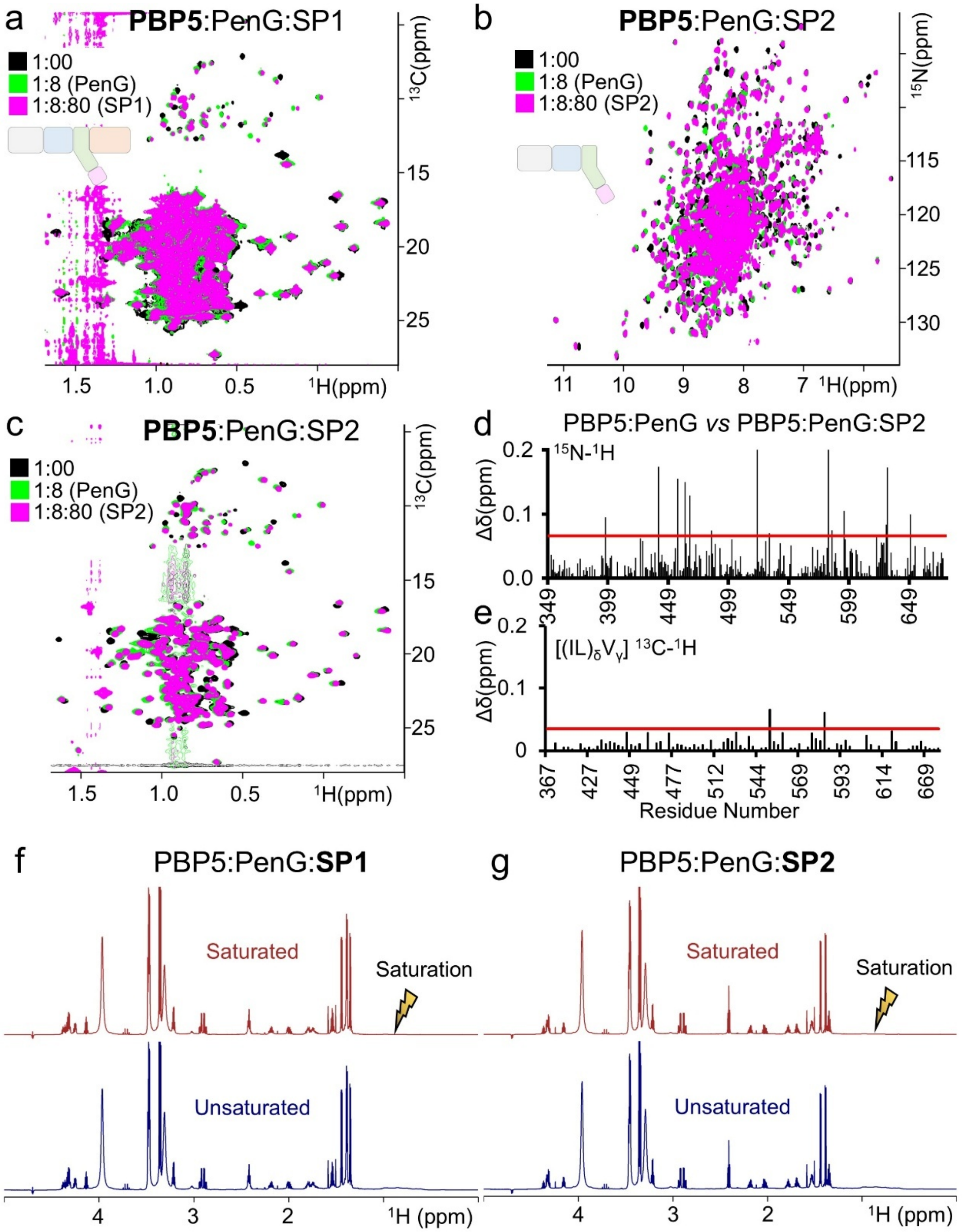

Supplementary Figure 6: Interaction of SP1 and SP2 with PBP5 in the presence of PenG. (**a**) Overlay of 2D [^{1}H,^{13}C] HMQC spectrum of PBP5 (black) saturated by PenG (1:8) (green) and titrated by SP1 (1:8:80; magenta). Overlay of 2D (**b**) [^{1}H,^{15}N] TROSY and (**c**) [^{1}H,^{13}C] HMQC spectrum of PBP5 (black) saturated by PenG (1:8) (green) and titrated by SP2 (1:8:80; magenta). (**d**) ^{1}H/^{15}N and (**e**) ^{1}H/^{13}C ILV methyl CSPs vs residue number plot for PBP5:PenG:SP2 (1:8:80 ratio); average+1σ (red line) for the PBP5 TP domain. (**f**) Overlay of saturation transfer difference (STD) NMR spectrum of PBP5 and SP1 at 1:100 ratio; red is saturated (0.73 ppm), and blue is reference (unsaturated, -40 ppm) spectrums. (**g**) Overlay of STD NMR spectra of PBP5 and SP2 at 1:100 ratio; red is saturated (0.73 ppm), and blue is reference (unsaturated, -40 ppm) spectrum.

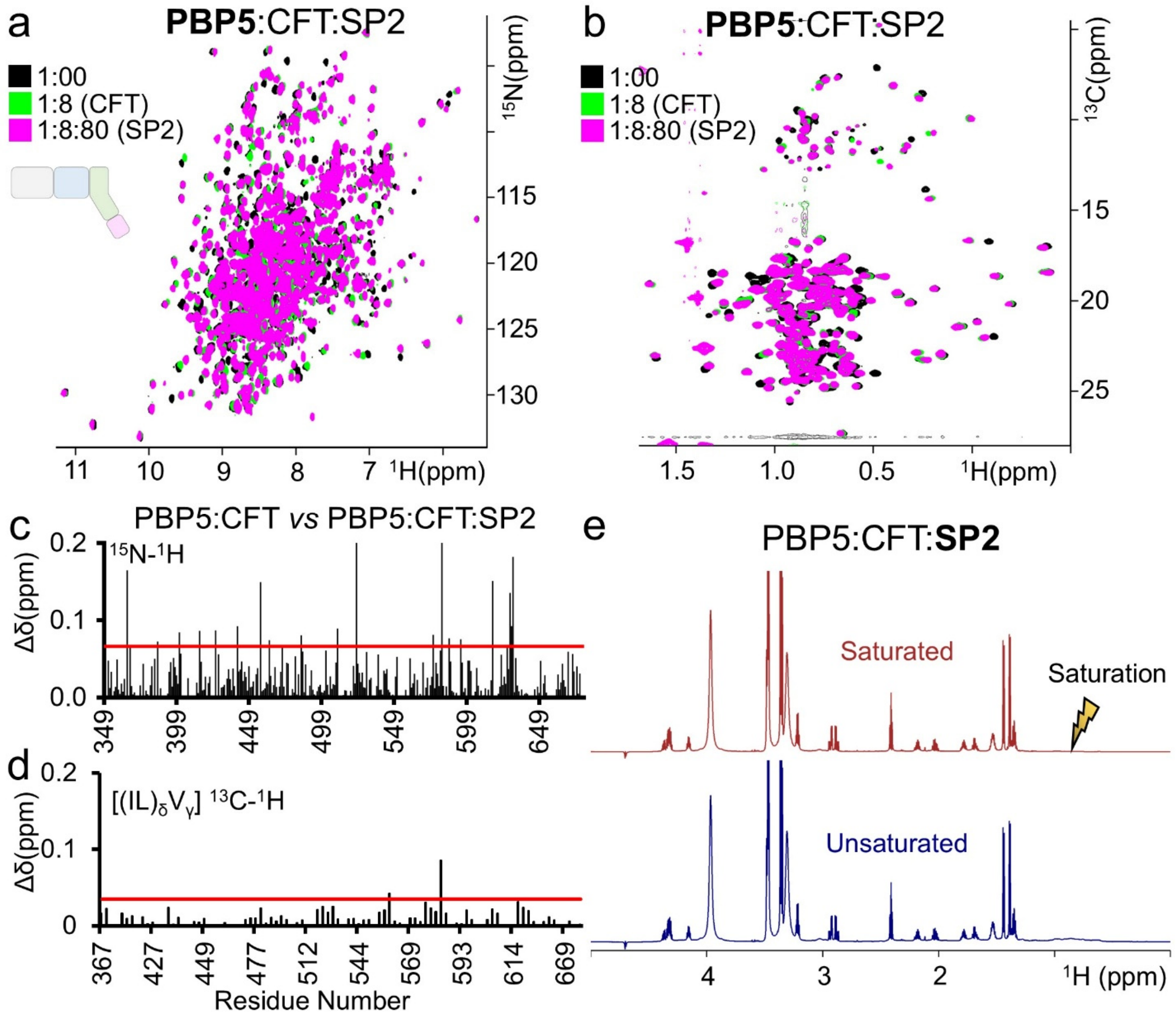

Supplementary Figure 7: Interaction of SP2 with PBP5 in the presence of ceftaroline. Overlay of 2D (**a**) [^{1}H,^{15}N] TROSY and (**b**) [^{1}H,^{13}C] HMQC spectrum of PBP5 (black) saturated by CFT (1:8) (green) and titrated by SP2 (1:8:80; magenta). (**c**) ^{1}H/^{15}N and (**d**) ^{1}H/^{13}C ILV methyl CSPs vs residue number plot for PBP5:CFT:SP2 (1:8:80 ratio); average+1σ (red line) for the PBP5 TP domain. (**e**) Overlay of the saturation transfer difference (STD) NMR spectra of PBP5:CFT and SP2 at 1:100 ratio; red is saturated (0.73 ppm), and blue is reference (unsaturated, -40 ppm) spectrum.

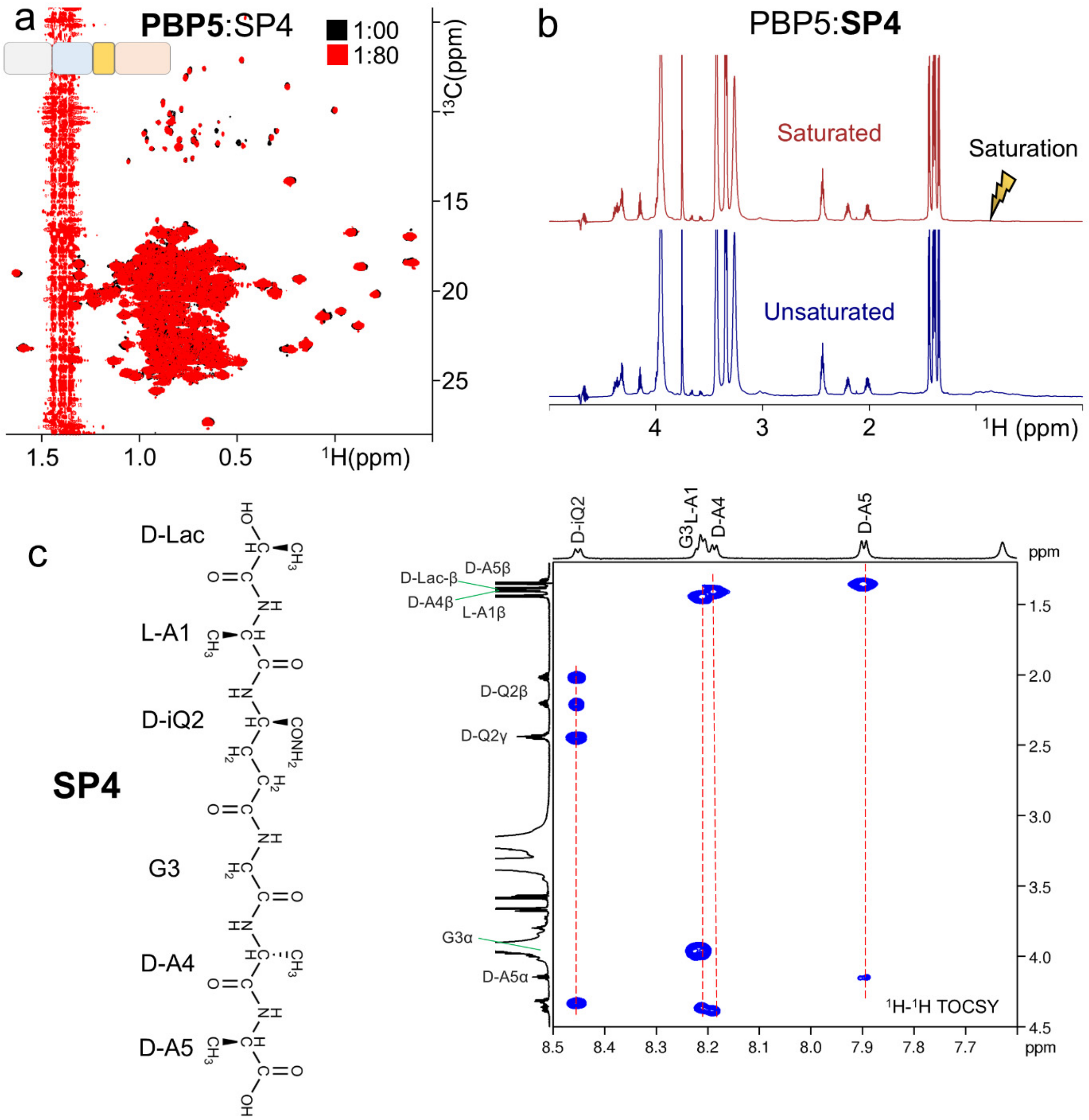

Supplementary Figure 8: D-Lys(D-iAsn) is necessary and sufficient for binding PBP5. (**a**) Overlay of 2D [^{1}H,^{13}C] HMQC spectrum of PBP5 (black) with SP4 (red) at 1:80 ratio. (**b**) Overlay of saturation transfer difference (STD) NMR spectrum of PBP5 and SP4 at 1:100 ratio; red is saturated (0.73 ppm), and blue is reference (unsaturated, -40 ppm) spectrums. (**c**) 2D [^{1}H,^{1}H] TOCSY spectrum of SP4 with the resonance assignments, chemical structure shown left.

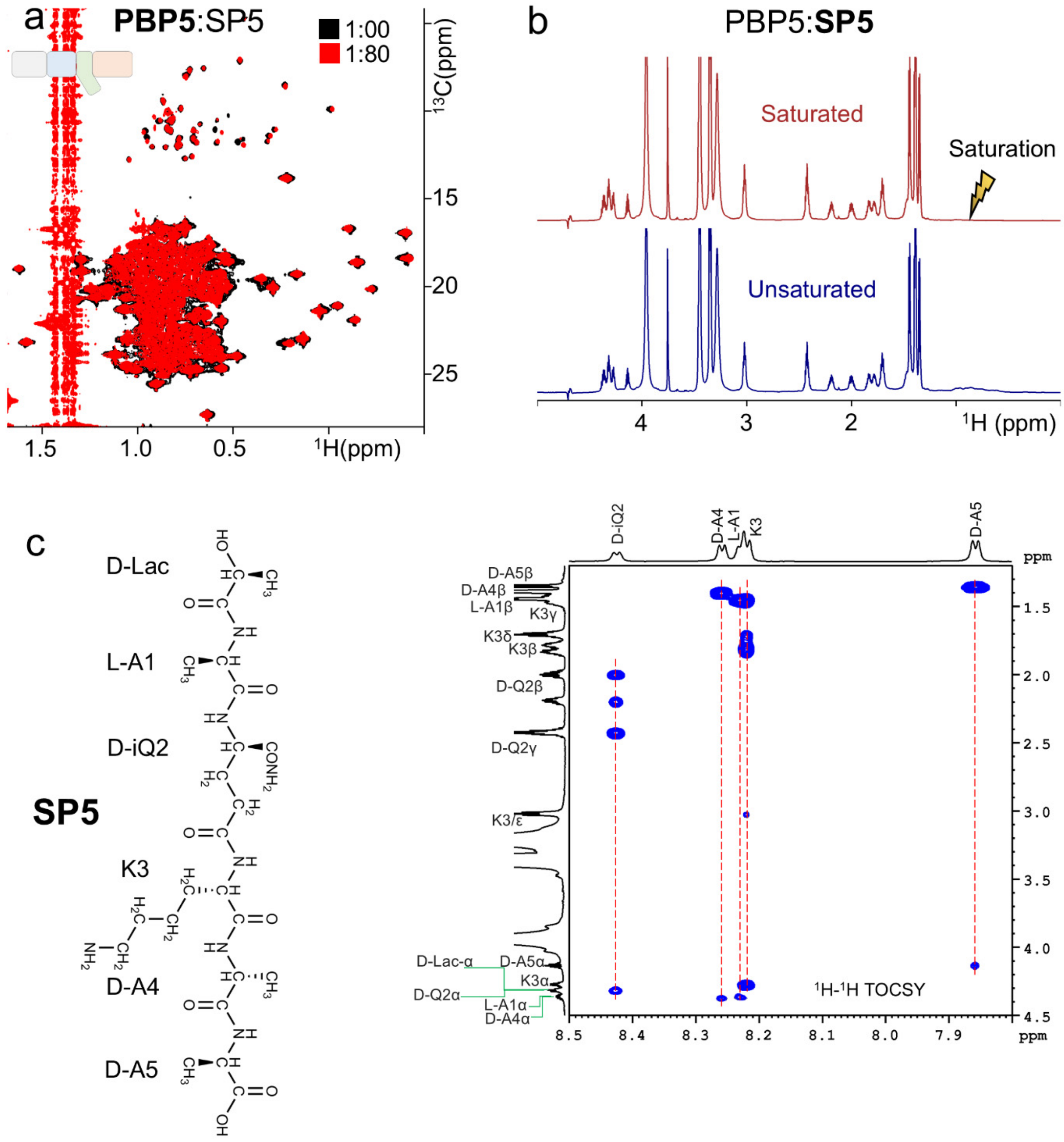

Supplementary Figure 9: D-iAsn contributes to PBP5 binding. (**a**) Overlay of 2D [^{1}H,^{13}C] HMQC spectrum of PBP5 (black) with SP5 (red) at 1:80 ratio. (**b**) Overlay of saturation transfer difference (STD) NMR spectrum of PBP5 and SP5 at 1:100 ratio; red is saturated (0.73 ppm), and blue is reference (unsaturated, -40 ppm) spectrums. (**c**) 2D [^{1}H,^{1}H] TOCSY spectrum of SP5 with the resonance assignments, chemical structure shown left.

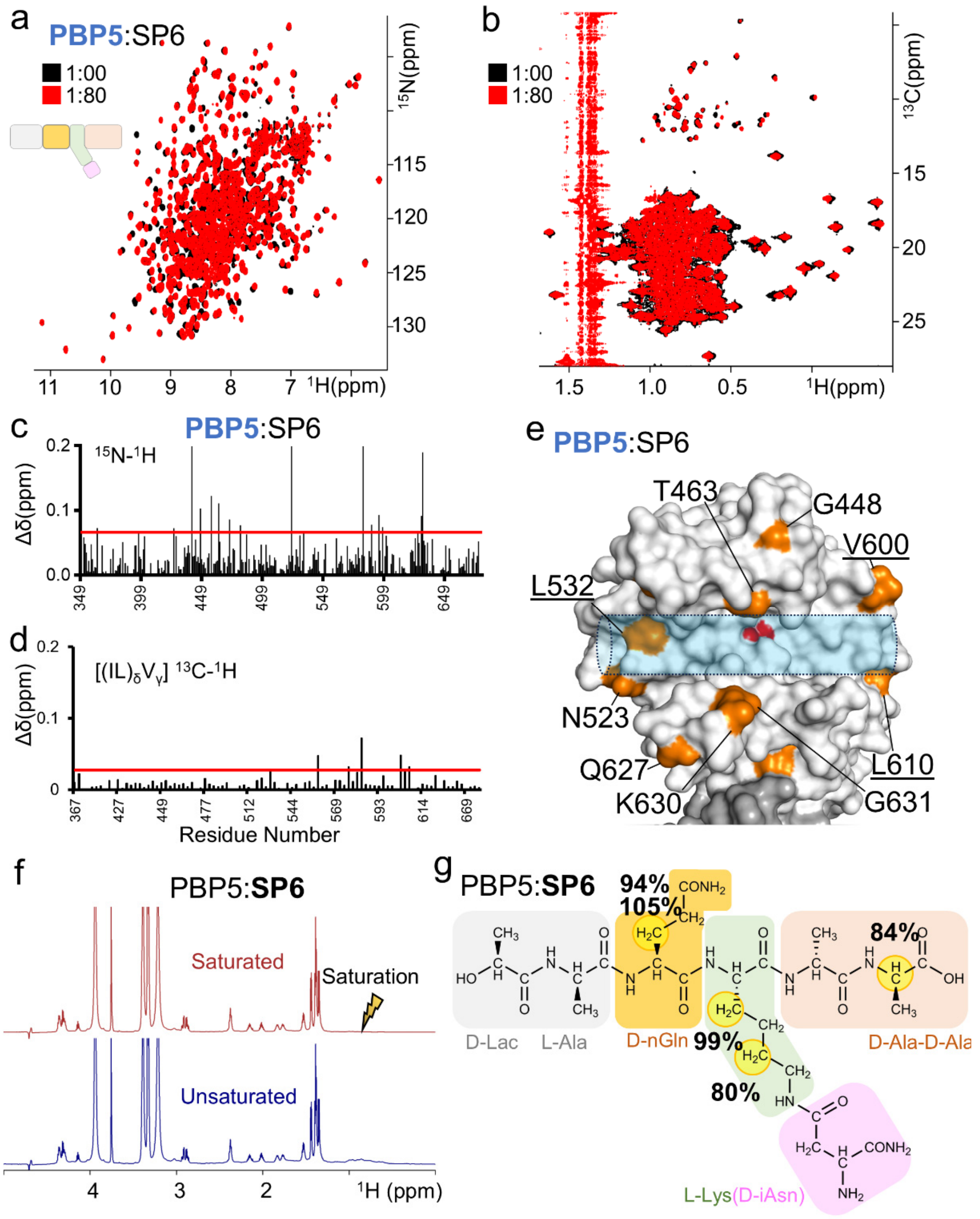

Supplementary Figure 10: **Changing the D-iGln to D-nGln does block PBP5 binding.** Overlay of 2D (**a**) [^{1}H,^{15}N] TROSY and (**b**) [^{1}H,^{13}C] HMQC spectrum of PBP5 (black) titrated by SP6 (1:80; red). (**c**) ^{1}H/^{15}N and (**d**) ^{1}H/^{13}C ILV methyl CSPs vs residue number plot for PBP5:SP6 (1:80); average+1σ (red line) for the PBP5 TP domain. (**e**) SP6 CSPs mapped on PBP5 structure (gray surface; PDBid: 6MKA); orange surface: residues with significant changes (underlined residues indicate ^{1}H/^{13}C ILV methyl data). S422 is highlighted in red. (**f**) Overlay of saturation transfer difference (STD) NMR spectrum of PBP5:SP6 at 1:100 ratio; red is saturated (0.73 ppm), and blue is reference (unsaturated, -40 ppm) spectrum. (**g**) Molecular structure of SP6, yellow circles highlight ^{1}H atoms with >50% STD enhancement.

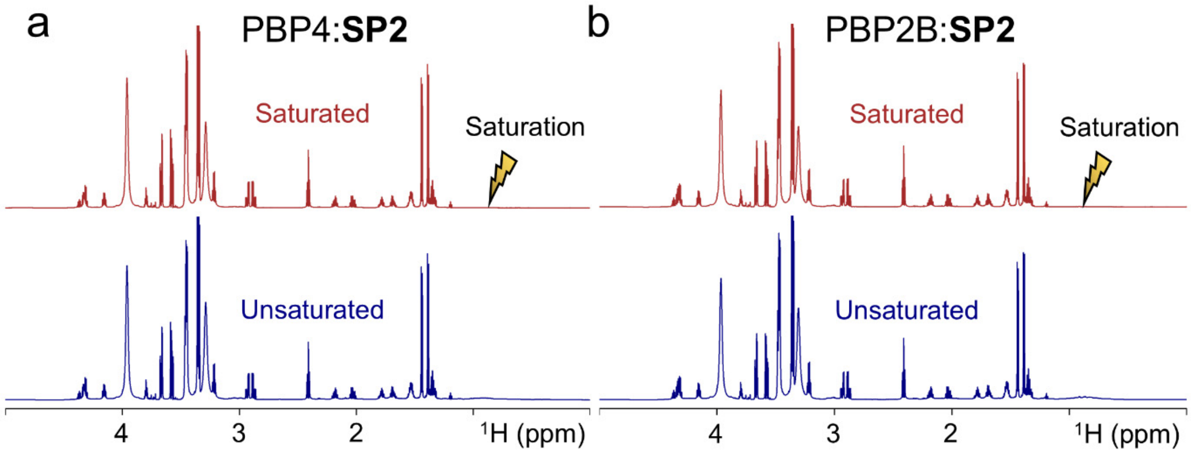

Supplementary Figure 11. STD experiments of SP2 interacting with PBP4 and PBP2B of *E. faecalis*. Overlay of STD NMR spectra of (**a**) PBP4:SP2 and (**b**) PBP2B:SP2 at 1:100 ratio; red is saturated (0.73 ppm), and blue is reference (unsaturated, -40 ppm) spectrum.

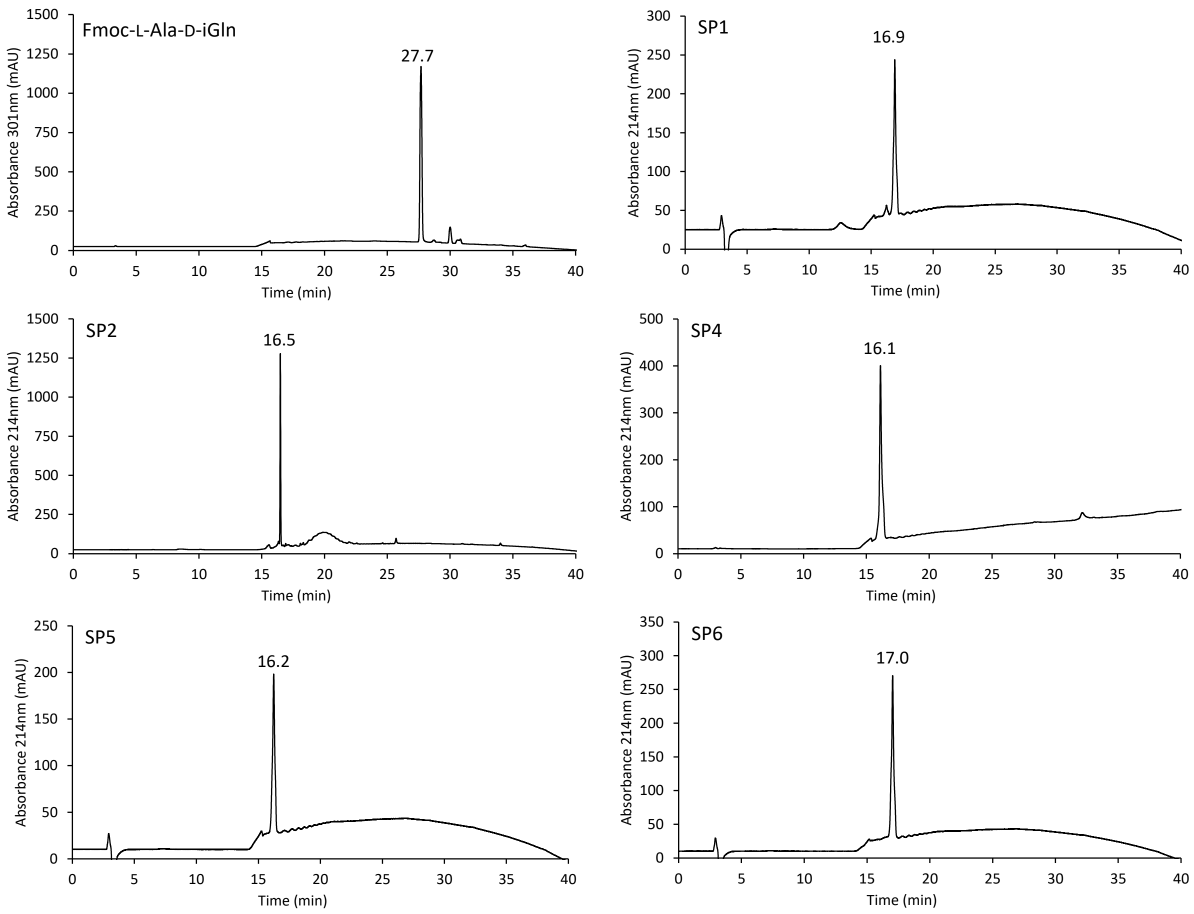

Supplementary Figure 12: **Analytical rpHPLC chromatograms of synthesized peptides**. Fmoc-L-Ala-DiGln, SP1, SP2, SP4, SP5 and SP6 are shown.

Supplementary Table 1: Binding affinity of PBP5 with SP1; residues with statistically significant changes are reported using NH chemical shift changes.

Residue Name	K_D (mM)
D472	4.4
N523	2.7
Y554	2.4
Q627	5.3
F636	3.2
R665	3.3
Q671	4.3
Y677	4.8
mean±std	3.9 ± 1.0

Supplementary Table 2: Disk diffusion assay results (all measurements n=3)

	Zone Size (mm) [Range]		
	**Ampicillin**	**Penicillin**	**Ceftriaxone**
Wild Type	32 [30-32]	28 [28-32]	20 [20-26]
pCWR624	0 [0]	0 [0]	0 [0]
pCWR624Δpbp5	30 [30-32]	28 [28-32]	24 [22-28]
pCWR624Δ*pbp5_pbp5*_V1	16 [14-16]	0 [0]	0 [0]
pCWR624Δ*pbp5_pbp5*_V2	26 [26-28]	26 [26-28]	24 [24-26]
pCWR624Δ*pbp5_pbp5*_V3	32 [30-32]	32 [30-32]	28 [26-28]
pCWR624Δ*pbp5_pbp5*_V4	32 [32]	32 [30-32]	26 [26-28]
pCWR624Δ*pbp5_pbp5*_V5	0 [0-10]	0 [0]	0 [0]
pCWR624Δ*pbp5_pbp5*_V6	0 [0-10]	0 [0]	0 [0]

Supplementary Table 3: MIC (all measurements n=3)

	MIC (µg/ml) [Range]		
	**Ampicillin**	**Penicillin**	**Ceftriaxone**
Wild Type	0.39 [0.39-0.78]	0.39 [0.39-0.78]	50 [25-100]
pCWR624	100 [100->200]	>200 [>200]	>3200 [3200->3200]
pCWR624Δpbp5	0.78 [0.39-1.56]	0.39 [0.39-1.56]	50 [25-100]
pCWR624Δ*pbp5_pbp5*_V1	100 [50-100]	200 [200]	3200 [1600-3200]
pCWR624Δ*pbp5_pbp5*_V2	6.25 [6.25-12.5]	12.5 [12.5-25]	50 [50-100]
pCWR624Δ*pbp5_pbp5*_V3	<0.19 [<0.19-0.39]	0.39 [0.39]	25 [25]
pCWR624Δ*pbp5_pbp5*_V4	<0.19 [<0.15-0.39]	0.39 [0.39]	25 [25-50]
pCWR624Δ*pbp5_pbp5*_V5	100 [100]	200 [200]	>3200 [>3200]
pCWR624Δ*pbp5_pbp5*_V6	100 [100]	200 [200]	>3200 [>3200]

Supplementary Table 4: Mass spectrometry of synthetized SP peptides.

Peptide	Sequence	Formula	Calculated [M]	Observed $[M+H]^+$
SP1	D-Lac-L-Ala-D-iGln-L-Lys(D-iAsn)-D-Ala-D-Ala	$C_{27}H_{47}N_9O_{11}$	673.34	674.47
SP2	D-Lac-L-Ala-D-iGln-L-Lys(D-iAsn)	$C_{21}H_{37}N_7O_9$	531.27	532.33
SP4	D-Lac-L-Ala-D-iGln-Gly-D-Ala-D-Ala	$C_{19}H_{32}N_6O_9$	488.22	488.82
SP5	D-Lac-L-Ala-D-iGln-L-Lys-D-Ala-D-Ala	$C_{23}H_{41}N_7O_9$	559.30	560.40
SP6	D-Lac-L-Ala-D-nGln-L-Lys(D-iAsn)-D-Ala-D-Ala	$C_{27}H_{47}N_9O_{11}$	673.34	674.47

Uncropped gel images

Figure 3

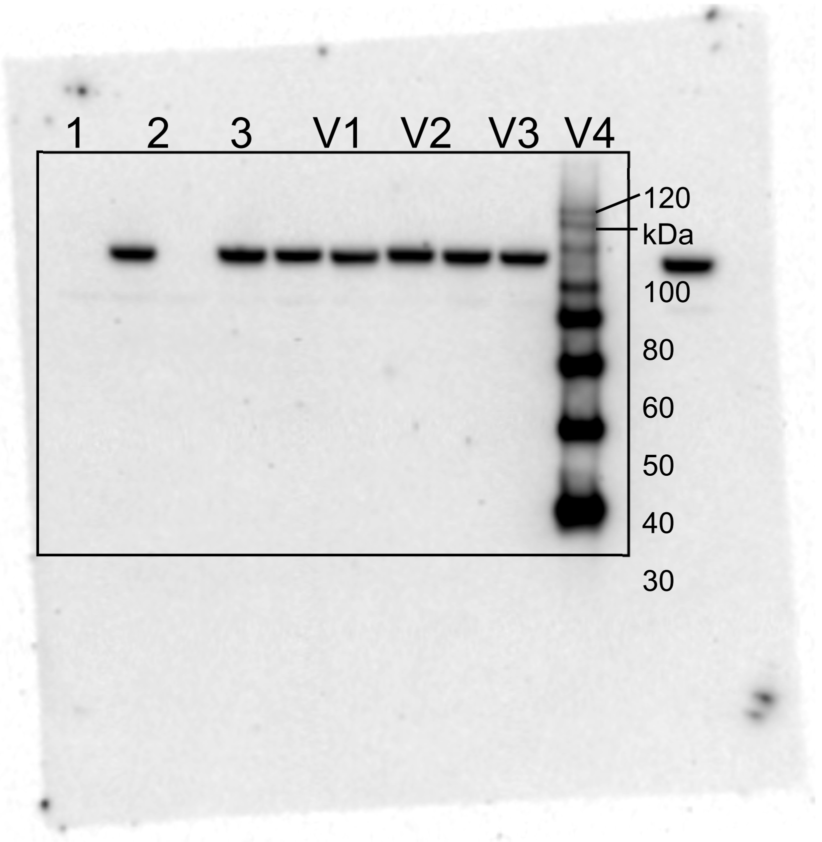

Supplemental Figure 3

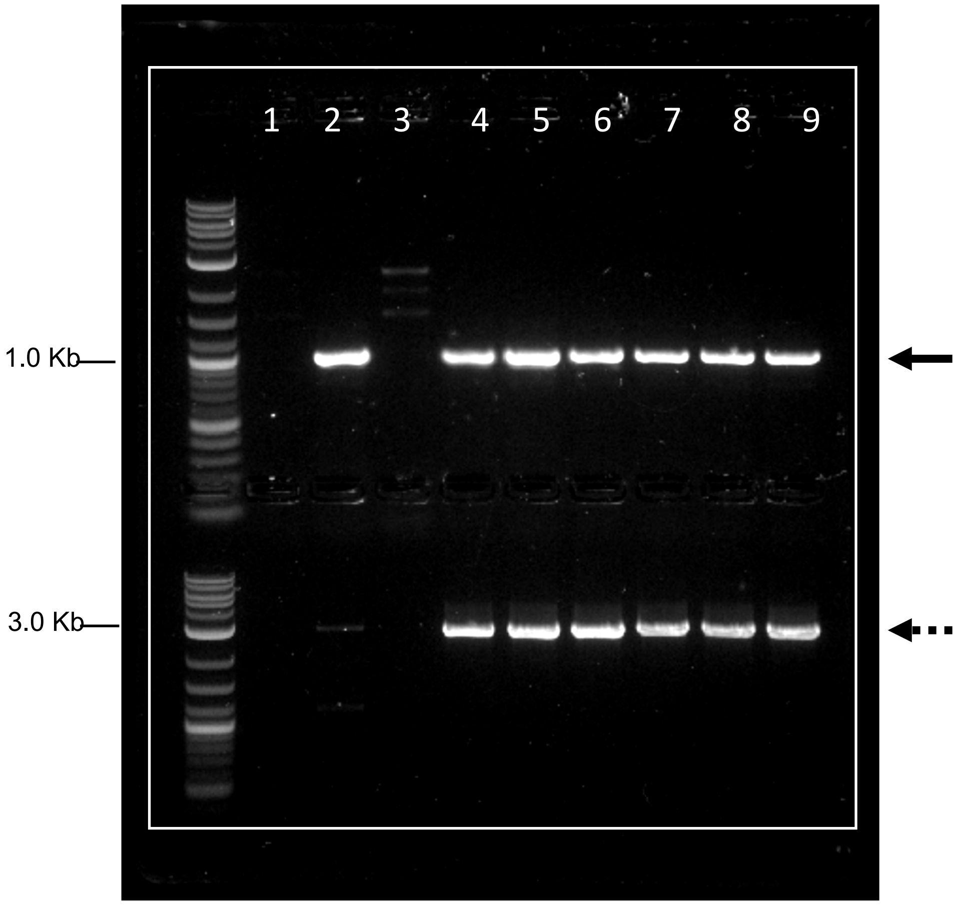

Supplemental Figure 3

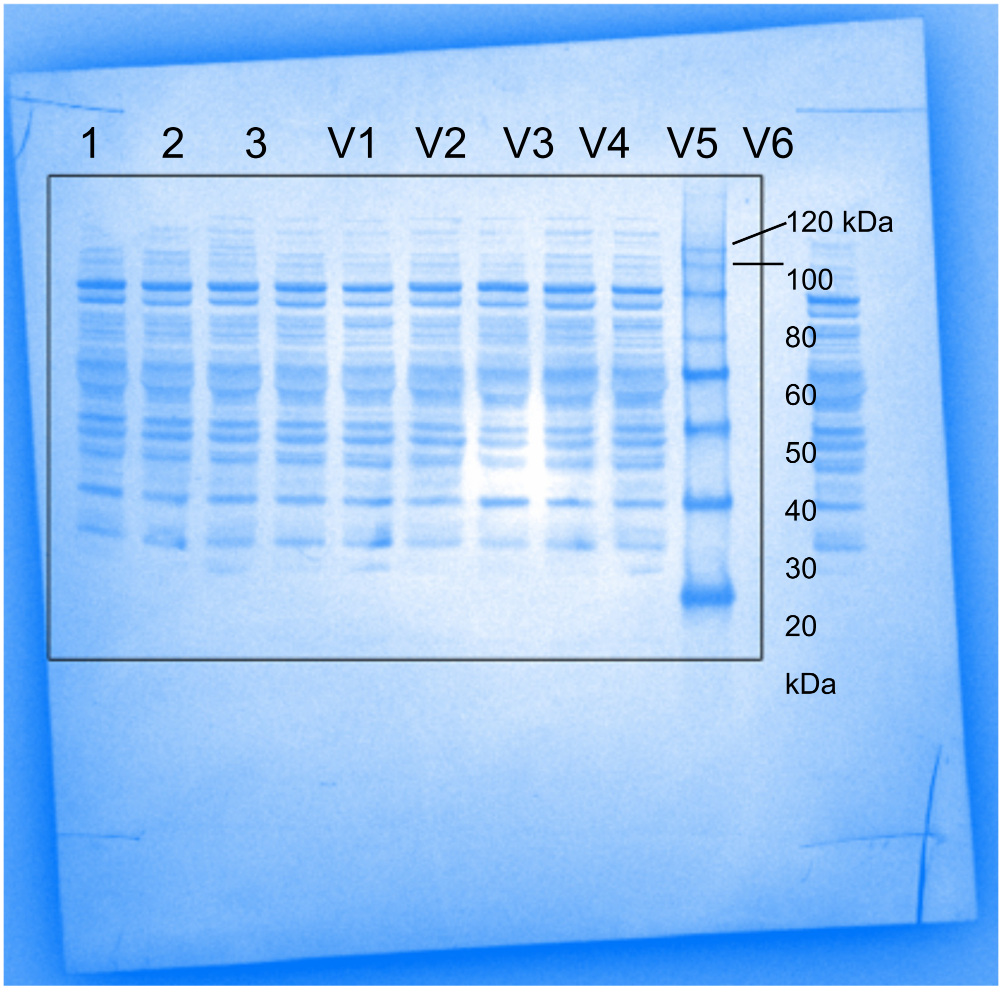

Supplement: Supplementary file 1 — Supplementary Information [file 41467_2025_66095_MOESM1_ESM.pdf]
